# How Hydrotropy Explains the Influence of Dissolved Gases on the Properties of Aqueous Salt Solutions

**DOI:** 10.1021/acs.jpcb.6c00378

**Published:** 2026-05-01

**Authors:** Eudes Eterno Fileti, Dinis O. Abranches, João A. P. Coutinho

**Affiliations:** † Instituto de Ciência e Tecnologia, Universidade Federal de São Paulo, 12247-014 São José dos Campos, SP, Brazil; ‡ CICECO − Aveiro Institute of Materials, Department of Chemistry, 426216 University of Aveiro, Aveiro 3810-193, Portugal

## Abstract

Dissolved atmospheric gases are typically neglected in models of aqueous electrolytes, yet several past examples in the literature reveal physicochemical property anomalies (e.g., changes in electrical conductivity) upon degassing. Here, we use classical molecular dynamics simulations to investigate whether dissolved nitrogen can reorganize the microscopic structure of 0.5 M potassium salt solutions (KX). These simulations closely mimic previous experimental work by Ninham and Lo Nostro, which reported unusual conductivity changes depending on whether dissolved gas was present. By comparing systems with and without N_2_ for a series of halide and molecular anions, radial distribution functions, coordination numbers, and spatial distribution functions reveal that N_2_ perturbs electrolyte structure through collective, hydrotropy-like solvent organization. Molecular anions with diffuse hydration shells display anisotropic gas-anion interactions and support weak spatial correlations of N_2_ molecules, whereas halides remain structurally rigid and largely insensitive to N_2_. Viewed in terms of hydrotrope-like aggregation between gas and anions, these results explain the conductivity anomalies reported in earlier experiments. Altogether, the effects on the conductivity due to the dissolved gas arise not from local kinetic changes but from mesoscale solvent structuring driven by gas-ion–water cooperativity, providing a molecular-level explanation for gas-mediated ion-specific phenomena in aqueous electrolytes.

## Graphical abstract

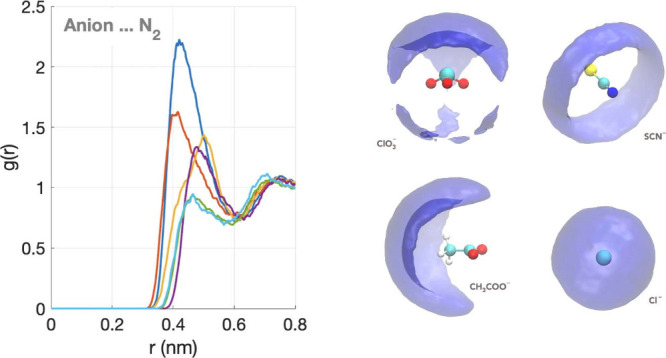

## Introduction

Dissolved atmospheric gases are usually treated as inert spectators in classical theories of liquids and electrolyte solutions. Their solubilities in water, particularly those of N_2_ and O_2_, are sufficiently low that most theoretical and computational frameworks simply neglect their presence.^1^ However, a growing body of experimental evidence demonstrates that dissolved gases can induce significant ion-specific effects in aqueous systems.^1,2^ Vogel et al.^2^ experimentally demonstrated that the presence of atmospheric CO_2_ leads to a pronounced decharging of dielectric surfaces in aqueous suspensions, far exceeding what would be expected from CO_2_ dissolution and the formation of its ionic species (HCO_3_^–^/CO_3_^2–^) alone. This effect substantially alters ion distributions and interfacial charge regulation near aqueous interfaces.^2^ Complementary evidence comes from interfacial vibrational sum-frequency spectroscopy (VSFS) studies, which show that dissolved CO_2_ modifies the O–H stretching bands of water and perturbs molecular-level interactions at the vapor–water interface, with direct consequences for interfacial properties governing the spatial organization of ions and molecules.^3^ More broadly, a large body of experimental work on ion-specific effects (SIEs), including Hofmeister phenomena, has demonstrated behaviors that cannot be accounted for by primitive electrostatic frameworks such as DLVO theory, particularly at water-vapor interfaces and in the presence of additional molecular species.^4^

Recent experiments by Ninham and Lo Nostro show that the removal of dissolved atmospheric gases (“degassing”) produces *unexpected, specific changes* in the electrical conductivity of aqueous potassium salt solutions.^1^ While all solutions behave similarly below ∼0.17 M, clear divergences emerge at higher concentrations. Potassium halides (KF, KCl, KBr, KI), classified as α/α electrolytes, exhibit a decrease in conductivity after degassing. In contrast, salts with a more amphiphilic nature, namely potassium acetate (KAc), chlorate (KClO_3_), and thiocyanate (KSCN), all classified as α/β pairs, show an increase in conductivity. These opposite responses mirror the same ion-specific patterns found in bubble–bubble coalescence and support the view that dissolved gases and nanobubble stabilization play an active role in concentrated electrolytes; precisely in the concentration regime of the systems investigated here.^1^

These reproducible anomalies, often described in terms of the so-called α/α or α/β character of the ion pair, seem to challenge conventional electrostatic theories and indicate that dissolved gases participate in subtle modes of organization within aqueous electrolytes.^1^ In Ninham’s framework, β-type ions are weakly hydrated and polarizable, tending to populate interfacial or low-density water regions, whereas α-type ions are strongly hydrated and remain tightly embedded in the bulk hydrogen-bond network. Electrolytes composed of two α-type ions (α/α) display diffuse solvation environments and show pronounced sensitivity to degassing, while α/β salts respond weakly, often in an opposite direction. The parallel between this classification and the observed conductivity changes points to a gas-mediated, anion-nitrogen aggregation mechanism rather than a simple electrostatic or kinetic effect.^1^

The observations discussed in the previous paragraphs are reminiscent of those seen in hydrotropic behavior. Thus, although Ninham’s interpretation emphasizes nanobubble formation, an alternative and conceptually related molecular mechanism based on hydrotropy may also be central to understanding these unexpected behaviors. Hydrotropy refers to the ability of certain amphiphilic molecules, known as hydrotropes, to enhance the aqueous solubility of hydrophobic, poorly soluble solutes. Its mechanism is based on hydrophobic-force-driven aggregation of hydrotropes and solutes. Briefly, the number of hydrogen bonds among water molecules is maximized when the apolar surfaces of the hydrotropes aggregate around the solute, leading to a stabler system and, thus, enhancing the solubility of the solute.^5,6^ Many common hydrotropes are amphiphilic salts (akin to those classified as α/β pairs), and it has been shown that hydrotropic efficiency (or, in other words, readiness to aggregate around a hydrophobic solute) is directly connected to how amphiphilic an ion is.^7^

The mechanism of aggregation of hydrotropy need not be restricted to classical organic hydrotropes: they can, in principle, extend to any species whose presence perturbs local water structure cooperatively,^8–11^ including dissolved gases. Weakly soluble gases such as N_2_ can, in principle, engage in similar cooperative interactions: in electrolyte solutions, the reorganization of water and ions may promote gas-ion aggregates analogous to solute-hydrotrope aggregates. Removing dissolved gas through degassing thus eliminates a weakly hydrophobic cosolute and shifts local solvation equilibria. Any resulting changes in conductivity are therefore most plausibly attributed to collective, solvent-mediated reorganization, rather than to direct gas–ion binding.

This hydrotropic interaction framework motivates the use of molecular dynamics (MD) simulations to probe whether dissolved N_2_ influences water and electrolyte structure in ways consistent with such cooperative organization. To explore this hypothesis at the molecular level, we performed classical molecular dynamics (MD) simulations for a series of aqueous potassium salt solutions, namely: aqueous potassium salt solutions, KX, where the anion X was varied among ClO_3_^–^, CH_2_COO^–^ (Ac), SCN^–^, I^–^, Br^–^, Cl^–^ and F^–^. Motivated by this hydrotropic perspective, we use classical molecular dynamics simulations to examine how dissolved N_2_ influences the organization of aqueous potassium salt solutions. Our analysis focuses on collective solvent responses; such as density fluctuations and gas–ion spatial correlations; rather than on direct gas–ion binding. The results should allow probing the perturbation that N_2_ induces on the solution, in particular to evaluate if it affects the short-range ion pairing or local hydration, or rather if it reorganizes the solvent in an anion-dependent manner. This is expected to provide information to rationalize the conductivity anomalies observed experimentally.

## Methodology

The structural analysis of the aqueous electrolytes was carried out using classical molecular dynamics (MD) simulations. Aqueous ∼0.5 M potassium salt solutions, KX, were prepared, where the anion X was varied among ClO_3_^–^, CH_2_COO^–^ (Ac), SCN^–^, I^–^, Br^–^, Cl^–^ and F^–^. Simulations were performed both in the absence and in the presence of dissolved N_2_ gas molecules.

All initial configurations were generated using PACKMOL,^12^ comprising 1500 water molecules, 15 KX ion pairs, and, for the gas-containing systems, an additional 10 nitrogen molecules. The elevated N2 count was chosen only to increase the statistical sampling of gas-ion and gas–water spatial correlations within tractable simulation times. Thus, while the N_2_ concentration employed is higher than experimental solubility, it enables statistically meaningful sampling of weak gas–ion correlations. This setup ensured a uniform spatial distribution of solute and solvent species throughout the simulation box. The intra- and intermolecular interactions were modeled using the SPC model for water^13^ and OPLS-AA force field^14^ for other species, incorporating nonadditive interaction potentials. These models are widely used and extensively benchmarked for aqueous electrolyte solutions, reproducing ion–water radial distribution functions, coordination numbers, and bulk properties with good reliability. Recent examples include applications to aqueous salts.^15–19^ Experimentally, densities of moderate-concentration potassium salt solutions at ambient conditions are very close to that of pure water (∼1.00–1.04 g cm^–3^), a range also reproduced by our simulations (1000–1050 kg m^–3^). This consistency supports the suitability of the chosen force fields for the present structural study.

Each system was first equilibrated and simulated in the isothermal–isobaric (*NPT*) ensemble at 1 atm and 300 K. The initial 2 ns of each trajectory were discarded as equilibration, and the subsequent 40 ns were used to obtain densities, cohesive energies, and radial distribution functions (RDFs). The equations of motion were integrated using a 1 fs time step. Electrostatic interactions were treated using the Coulomb law with a real-space cutoff of 1.2 nm, while long-range electrostatics were computed using the Particle Mesh Ewald (PME) method.^20^ van der Waals interactions were modeled by a Lennard-Jones 12–6 potential, smoothly shifted to zero between 1.1 and 1.2 nm following the classical shifted-force scheme. Temperature control was achieved via the Bussi–Donadio–Parrinello velocity-rescaling thermostat^21^ (time constant of 0.1 ps), ensuring proper canonical ensemble sampling, while pressure was maintained by the Parrinello–Rahman barostat^22^ (time constant of 1.0 ps, compressibility of 4.5 × 10^–5^ bar^–1^).

All MD trajectories were propagated using the GROMACS simulation package.^23^ Structural properties, including radial and spatial distribution functions, were analyzed with the GROMACS utility tools and the TRAVIS program,^24^ where applicable.

## Results and Discussion

Figure 1 summarizes the mass densities and cohesive energy densities of the potassium salt solutions, with and without dissolved N_2_. These properties offer an initial structural view of how the electrolyte responds to gas insertion. Mass density reflects liquid compactness and is influenced by ion hydration, packing efficiency, and excluded-volume effects from solutes or gases. Cohesive energy density, defined as the vaporization energy per unit volume (see definition at figure caption), indicates the strength of intermolecular attraction. In electrolyte solutions, it arises from the balance among ion–water, water–water, and ion–ion interactions, which can be weakened or reorganized by hydrophobic N_2_.

**Figure 1:**
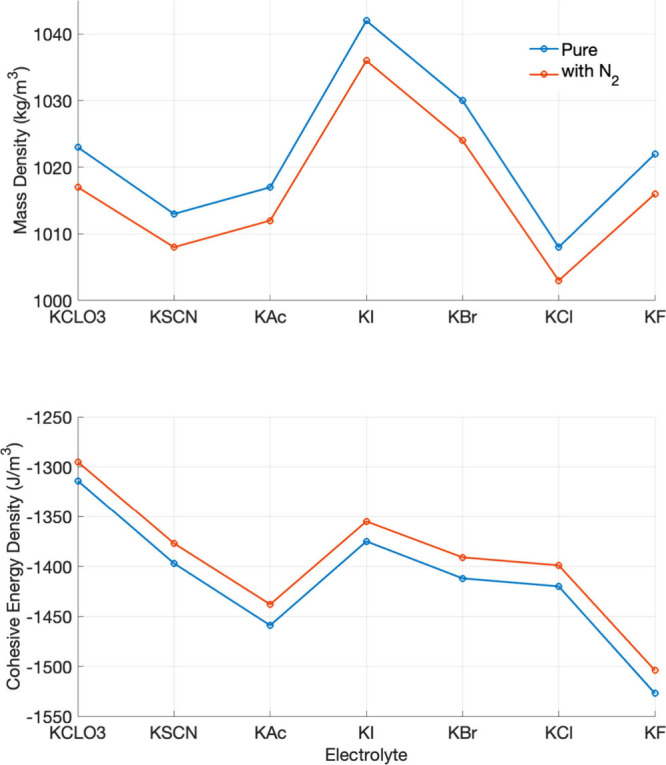
Mass density (in kg m^–3^) and cohesive energy density (in kJ mol^–1^ nm^–3^) of 0.5 M aqueous potassium salt solutions (KX), where X = ClO_3_^–^, SCN^–^, CH_2_COO^–^ (KAc), I^–^, Br^–^, Cl^–^ and F^–^. The blue curves correspond to the neat (N_2_-free) aqueous electrolytes, while the red curves correspond to electrolytes saturated with dissolved N_2_ gas, highlighting a slight decrease in density with N_2_. The bottom subplot presents the cohesive energy density (kJ mol^–1^ nm^–3^) for the same electrolytes, also showing a reduction in the presence of N_2_. The cohesive energy was calculated according to the equation *E*_*cohesive*_ = (*E*_*electrolyte*_ – *N*_1_ *E*_*ion pair*_ – *N*_2_ *E*_*water*_ – *N*_3_ *E*_*nitrogen*_)/*V*_*box*_. In this equation *E*_*cohesive*_ is the cohesive energy, *E*_*electrolyte*_ and *V*_*box*_ are the potential energy and volume of the electrolytes, *N*_1_, *N*_2_ and *N*_3_ are the numbers of ion pair, water, and nitrogen molecules, respectively. The reported energies are potential energies extracted directly from the MD gas phase (isolated molecule) trajectories. These are classical force-field potential energies, not electronic structure energies and not free energies. The magnitude of the standard deviations for all densities and energies was below 8 kg m^–3^ and 13 kJ mol^–1^ nm^–3^, respectively.

Across the salt series, the simulated mass densities display systematic variations that can be rationalized in terms of anion size, hydration strength, and polarizability. Electrolytes containing larger and more polarizable anions (e.g., I^–^, Br^–^) exhibit higher densities, whereas strongly hydrated, kosmotropic anions (e.g., F^–^, Cl^–^) lead to lower densities due to their more rigid and voluminous hydration shells. Molecular anions fall between these limits, reflecting their anisotropic shapes and intermediate hydration characteristics. Upon addition of dissolved N_2_, however, the change in mass density is essentially identical for all electrolytes. The density curves for systems with and without N_2_ display a nearly constant vertical offset of approximately 4–5 kg m^–3^, independent of anion identity. This uniform decrease is consistent with a simple excluded-volume effect associated with the presence of nitrogen molecules in the simulation box and does not reflect ion-specific modifications of hydration structure. A similar behavior is observed for the cohesive energy density. While absolute values vary across the salt series due to differences in ion–water and ion–ion interactions, the reduction induced by N_2_ is essentially uniform and falls within the statistical uncertainty of the simulations. Any small deviations between electrolytes are not systematic and cannot be interpreted as robust ion-specific effects.

The combined analysis of the radial distribution functions (RDFs) and coordination numbers (CNs), as reported in Figure 2, provides complementary insight into short-range ionic organization and into the spatial affinity between dissolved gas molecules and specific anions; an aspect central to evaluating whether hydrotropy-like mechanisms may play a role. The position of the first RDF peak shifts systematically across the series, moving to larger distances for larger and more polarizable halides (I^–^, Br^–^) and to shorter or more diffuse distances for strongly hydrated or structurally anisotropic anions such as F^–^ and the molecular ions SCN^–^, CH_3_COO^–^, and ClO_3_^–^. In the latter cases, broader peaks reflect the coexistence of multiple ion-pair geometries and a higher prevalence of solvent-shared configurations. Despite these clear ion-specific structural differences, the introduction of dissolved N_2_ has no measurable effect on the cation–anion RDFs. For all electrolytes, the RDFs obtained with and without N_2_ are virtually identical in peak position, height, and width, and the corresponding coordination numbers remain unchanged within statistical uncertainty. This demonstrates that dissolved nitrogen does not perturb short-range ion pairing or local electrostatic organization. Consequently, any influence of N_2_ on macroscopic properties, such as conductivity anomalies, cannot be attributed to modifications of direct cation–anion interactions, but must instead arise from more collective, solvent-mediated mechanisms.

**Figure 2:**
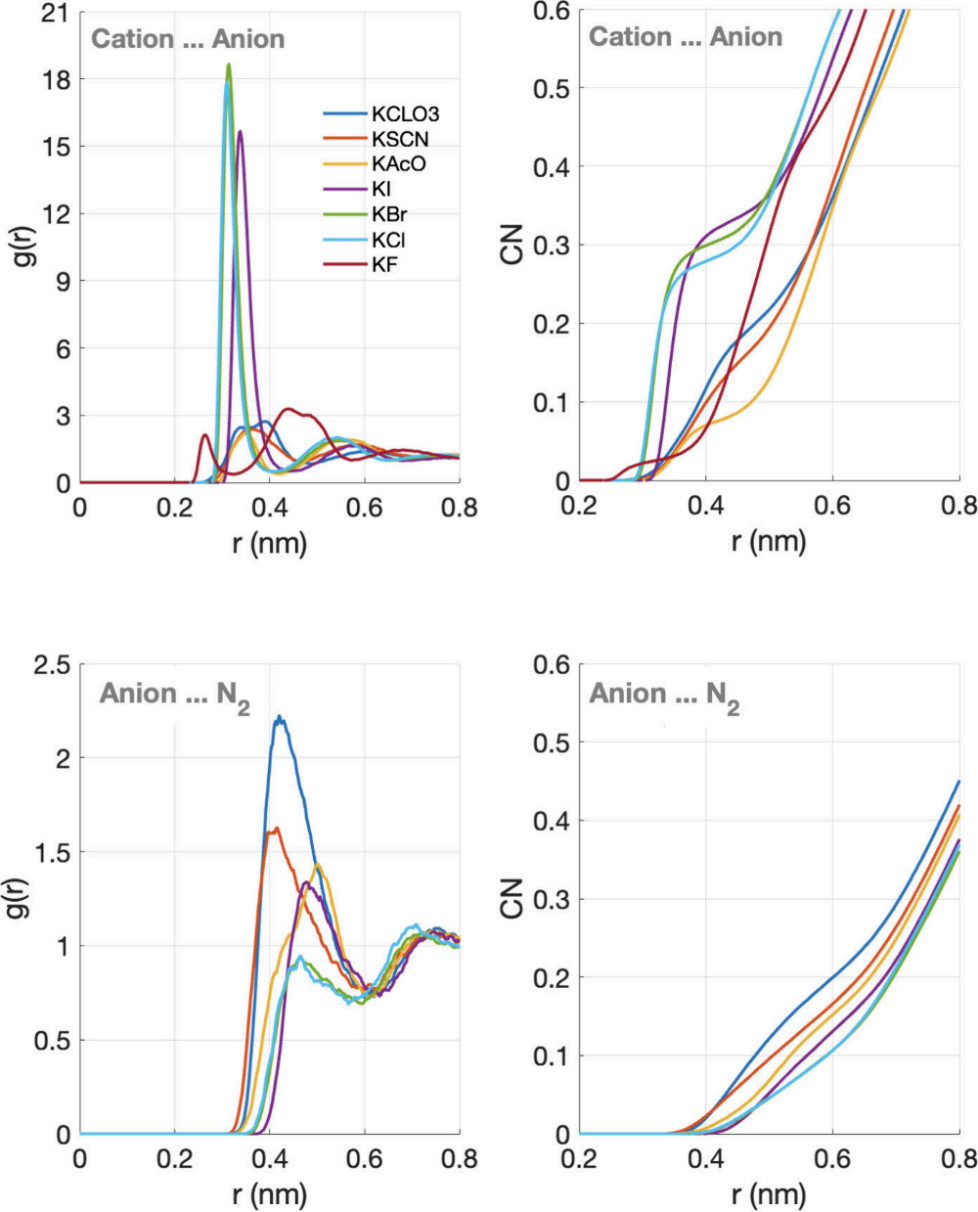
Center-of-mass radial distribution functions, *g*(*r*), and the corresponding coordination number, CN, for cation–anion and anion-water in 0.5 M aqueous potassium salt solutions (KX), where X = ClO_3_^–^, SCN^–^, CH_2_COO^–^ (KAc), I^–^, Br^–^, Cl^–^ and F^–^ obtained from molecular dynamics simulations. The curves correspond to simulations containing dissolved N_2_ molecules. Top-left: cation–anion correlations, showing the spatial arrangement and ion pairing strength for each salt. Top-right: cation–anion coordination number. Bottom-left: anion–N_2_ correlations, reflecting the strength and geometry of the hydrogen bonding between anions and water molecules. Bottom-right: anion–N_2_ coordination number.

In contrast, the anion-N_2_ RDFs provide essential information regarding the preferential localization of nitrogen relative to each anion. For the molecular and amphiphilic anions (SCN^–^, CH_3_COO^–^, and ClO_3_^–^), the RDFs exhibit a broad first maximum in the range of ∼0.35–0.45 nm with *g*(*r*) values slightly exceeding unity, indicating a weak but reproducible preferential interaction between N_2_ and these anions. In contrast, compact and strongly hydrated halides display flatter RDFs with *g*(*r*) ∼ 1 or slightly below, consistent with preferential exclusion of N_2_ from their immediate solvation environment. This dichotomy demonstrates that N_2_ is not uniformly distributed in the electrolyte but instead shows ion-specific preferences that correlate with anion amphiphilicity and hydration structure. The corresponding anion-N_2_ coordination numbers remain small (CN ≈ 0.17–0.21), confirming that these interactions are weak and nonstoichiometric; nevertheless, their systematic variation across the anion series provides clear evidence of preferential association versus exclusion behavior. Importantly, these effects do not reflect direct binding between N_2_ and the ions, nor do they perturb the short-range ionic structure, as confirmed by the invariance of cation–anion RDFs. Rather, the anion-N_2_ RDFs identify where nitrogen is statistically more or less likely to reside within the heterogeneous solvation landscape imposed by different ions. Such weak, ion-specific redistribution of dissolved gas is consistent with hydrotropy-like, solvent-mediated organization mechanisms and may represent the microscopic origin of the conductivity anomalies reported in degassing experiments.

The coordination numbers extracted from the anion-N_2_ RDFs reinforce this interpretation. The coordination number (CN) was calculated via standard integration of the RDF:

$$CN\left(r\right)=4\pi \rho \int_{0}^{r}g\left(r^{\prime }\right)r^{\prime 2}\;dr^{\prime }$$

where ρ is the bulk number-density of N_2_. By definition, CN­(r) increases monotonically with r because it is a cumulative integral. The physically meaningful CN values correspond to integration up to the first minmum of the RDF (i.e., the boundary of the first coordination shell). The absolute CN values shown here correspond to this first shell integration limit. While the absolute values remain small (∼0.17–0.21), their ion-specific variations indicate that N_2_ is not uniformly distributed in the electrolyte. The largest anion-N_2_ coordination is observed for ClO_3_^–^ (CN = 0.21), followed by SCN^–^ and CH_3_COO^–^ (CN = 0.17), whereas all halide anions (F^–^, Cl^–^, Br^–^, I^–^) exhibit uniformly low coordination numbers (CN = 0.04). This ordering reflects the greater amphiphilic character and more deformable hydration environments of molecular anions compared to the compact, strongly hydrated halides. Interestingly, this qualitative ranking mirrors the experimental conductivity trends reported by Ninham for 0.5 M solutions.1 Salts containing molecular anions (acetate, SCN^–^, ClO_3_^–^) show either small increases or negligible changes in conductivity upon degassing, whereas halide salts consistently exhibit conductivity reductions. While the absolute magnitude of the anion–N_2_ coordination remains small, well below one nitrogen molecule per anion, its ion-specific variation suggests that N_2_ is not uniformly distributed in solution, but preferentially associates with electrolytes capable of supporting heterogeneous solvent environments. Importantly, this correspondence is qualitative rather than quantitative: the modest differences in coordination number are insufficient to directly account for the magnitude of the conductivity anomalies. Instead, the data support a picture in which the anion-dependent localization of N_2_ acts as a structural marker of broader solvent reorganization. In this sense, the anion-N_2_ coordination reflects the propensity of certain electrolytes to accommodate gas molecules within perturbed hydration regions, consistent with collective, hydrotropy-like or mesoscale mechanisms rather than with direct, local binding or changes in short-range ionic structure.

The spatial distribution functions (SDFs) of N_2_ around the different anions reveal weak, anisotropic, and ion-dependent localization patterns that can be naturally interpreted within a hydrotropy-like framework (Figure 3).^8–11^ Because N_2_ is unable to engage in significant electrostatic or hydrogen-bonding interactions, its presence primarily perturbs the surrounding water network. Water molecules therefore tend to reorganize so as to maximize hydrogen bonding among themselves, effectively expelling N_2_ from highly structured, strongly hydrated regions and favoring its localization in more weakly constrained, low-connectivity environments. For molecular anions such as CH_3_COO^–^, ClO_3_^–^, and SCN^–^, the electrostatic potential maps show extended apolar or low-charge regions: in ClO_3_^–^, the axial region opposite the oxygen lone pairs is significantly less negative; in SCN^–^, the carbon–sulfur axis presents a narrow, elongated low-charge region; in CH_3_COO^–^, the methyl group forms a broad positive/neutral σ-region. In all these cases, the SDFs show that N_2_ preferentially occupies exactly these electrostatically mild, weakly structured regions, avoiding the strongly negative oxygen-rich areas where water hydrogen bonding is dominant. Thus, the anisotropic N_2_ localization arises not from chemical specificity or hydrophobic aggregation, but from the shape of the electrostatic potential field, which channels N_2_ into regions where interaction energy is least unfavorable. This interpretation is consistent with the coordination numbers (CN ≈ 0.17–0.21), which demonstrate that N_2_ does not bind to the anions, nor does it form stable complexes; instead, its presence is limited to transient, probabilistic occupation of electrostatically accessible volumes around the solute. Such small coordination number values, far below unity, confirm that these SDF features reflect weak spatial correlations preferences rather than “gas-rich” clustering.

**Figure 3:**
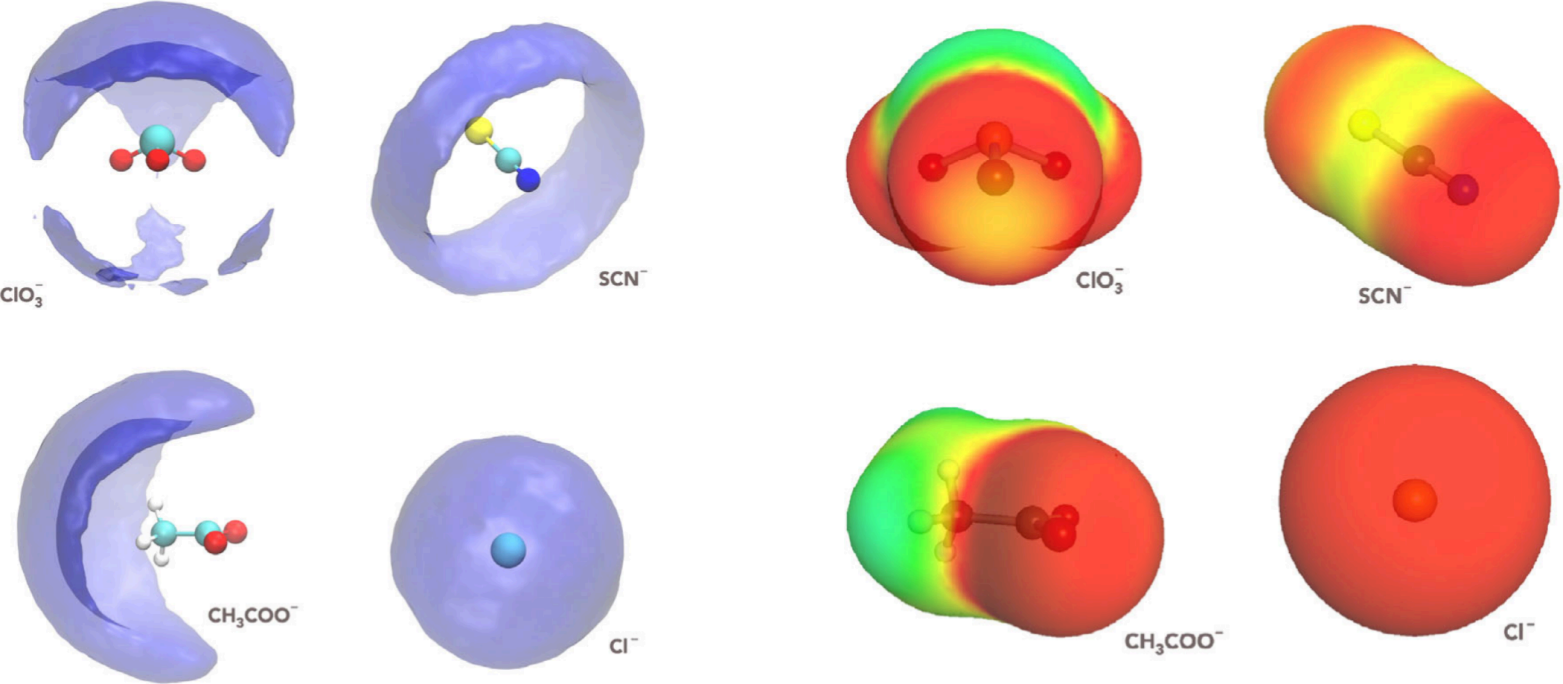
At left, spatial distribution functions (SDFs) of N_2_ molecules around different anions (ClO_3_^–^, SCN^–^, CH_3_COO^–^, and Cl^–^). The isosurfaces are normalized to represent a uniform density distribution, highlighting the preferential orientations and spatial arrangements of N_2_ molecules around each anion. At right, sigma surfaces of the anions, where the color scale represents the local surface charge density, ranging from more negative (red) to less negative or slightly positive regions (green/yellow). These sigma surfaces reveal anisotropies in surface polarity and amphiphilicity that correlate with the preferential localization of N_2_ observed in the SDFs. The SDFs and sigma surfaces for spherical anions such as F^–^, Br^–^, and I^–^ are omitted, as their isotropic distributions, similar to that of Cl^–^, lack directional preferences.

The present results indicate that N_2_ does not modify direct ion pairing but instead induces weak, anion-nitrogen aggregation. However, obtaining reliable dynamical observables from molecular dynamics simulations (particularly transport coefficients and time-correlation functions) requires significantly longer trajectories, larger system sizes, and multiple independent replicas to ensure proper statistical convergence. Even so, we analyzed two representative subsystems to explore the effect of dissolved N_2_ on transport properties, providing complementary dynamical insight. As shown at Table S2, KAc shows a conductivity decrease from ∼4.4 to 3.8 S m^–1^ upon N_2_ saturation, accompanied by a reduction in anion diffusion from ∼(1.5 to 1.2) × 10^–5^ cm^2^ s^–1^, consistent with the expected α/β behavior. In contrast, KF exhibits an increase in conductivity from ∼3.8 to 4.5 S m^–1^, together with a slight increase in anion diffusion from ∼(1.5 to 1.6) × 10^–5^ cm^2^ s^–1^, in line with α/α systems. These two cases were selected because they are the clearest representatives of the amphiphilic α/β and strongly hydrated α/α classes, respectively. Although these transport estimates are exploratory, they are consistent with the structural picture obtained from RDF, CN, and SDF analyses and provide complementary support for the proposed microscopic interpretation.

## Conclusions

The molecular dynamics results presented here provide a coherent microscopic picture of how dissolved nitrogen modulates the structure of aqueous electrolyte solutions and how these effects relate to the extraordinary conductivity anomalies reported experimentally. A central finding of this work is that the influence of N_2_ manifests primarily through anion-nitrogen aggregation and mesoscale gas structuring, rather than through direct perturbations of short-range hydration or hydrogen-bond kinetics.

The combined RDF, CN, and SDF analyses reveal a clear distinction between molecular anions (ClO_3_^–^, SCN^–^, CH_3_COO^–^) and halides (F^–^, Cl^–^, Br^–^, I^–^). Molecular anions possess diffuse, flexible, and less tightly bound hydration shells, which facilitate anisotropic interactions with N_2_ and the formation of transient gas-rich domains. Degassing therefore disrupts these domains and leads to significant rearrangements in hydration and ion pairing; consistent with the strong ion-specific conductivity changes observed experimentally.

By contrast, halides exhibit compact, isotropic hydration environments that tightly anchor surrounding water molecules. Their RDFs and CNs remain largely unchanged in the presence or absence of N_2_, their SDFs show only uniform distributions of gas, and their hydrogen-bond lifetimes reflect intrinsic ion properties rather than gas-mediated perturbations. Such structural rigidity explains why halides show comparatively weak conductivity anomalies upon degassing: they are simply less susceptible to gas-driven cooperative effects.

In summary, this study shows that dissolved nitrogen can modulate electrolyte properties through hydrotropy-like cooperative structuring, especially in systems with molecular anions and diffuse hydration shells. These findings provide microscopic evidence that helps reconcile simulation results with Ninham’s experimentally observed conductivity anomalies, strengthening the view that gas-ion–water coupling and mesoscale organization are central to the physics of concentrated aqueous electrolytes.

## Supplementary Material



## Notes

Additional structural and dynamical data, including composition of the systems, additional radial distribution functions, and exploratory dynamical properties (PDF)
