# Supplementary material for: How Hydrotropy Explains the Influence of Dissolved Gases on the Properties of Aqueous Salt Solutions
Source: J Phys Chem B. 2026 May 1;130(19):5059–64. doi: 10.1021/acs.jpcb.6c00378 (PMC13181767; doi:10.1021/acs.jpcb.6c00378)
Supplement: Supplementary file 1 [file jp6c00378_si_001.pdf]

SUPPORTING MATERIAL

How Hydrotropy Explains the Influence of Dissolved Gases on the Properties of Aqueous Salt Solutions

Eudes Eterno Fileti,[1] Dinis O. Abranches[2] and João A. P. Coutinho[2]

[1] Instituto de Ciência e Tecnologia, Universidade Federal de São Paulo, 12247-014, São José dos Campos, SP, Brazil

[2] CICECO − Aveiro Institute of Materials, Department of Chemistry, University of Aveiro, Aveiro 3810-193, Portugal

Table S1: Composition of the simulated systems. For every simulated system, the number of species (water, K^+X^- pair, N_2), the average box volume (nm^3), number of atoms at each box.

**System**	**Water**	**Ion pais**	**N_2**	**Box Volume (nm^3)**	**# Atoms**	**Concentration (M)**
**With N_2**						
$KCLO_3$	1500	15	10	47.60	4595	0.523
KSCN	1500	15	10	47.40	4580	0.525
KAcO	1500	15	10	47.22	4640	0.528
KI	1500	15	10	47.73	4550	0.521
KBr	1500	15	10	47.16	4550	0.528
KCl	1500	15	10	47.04	4550	0.529
KF	1500	15	10	46.03	4550	0.541
**Without N_2**						
$KCLO_3$	1500	15	0	46.87	4575	0.468
KSCN	1500	15	0	46.68	4560	0.474
KAcO	1500	15	0	46.50	4620	0.480
KI	1500	15	0	47.01	4530	0.464
KBr	1500	15	0	46.44	4530	0.482
KCl	1500	15	0	46.32	4530	0.486
KF	1500	15	0	45.31	4530	0.519

Concentration computed as: $[species]\ (M) = \frac{N_{species}/N_A}{V_{box}(L)}$

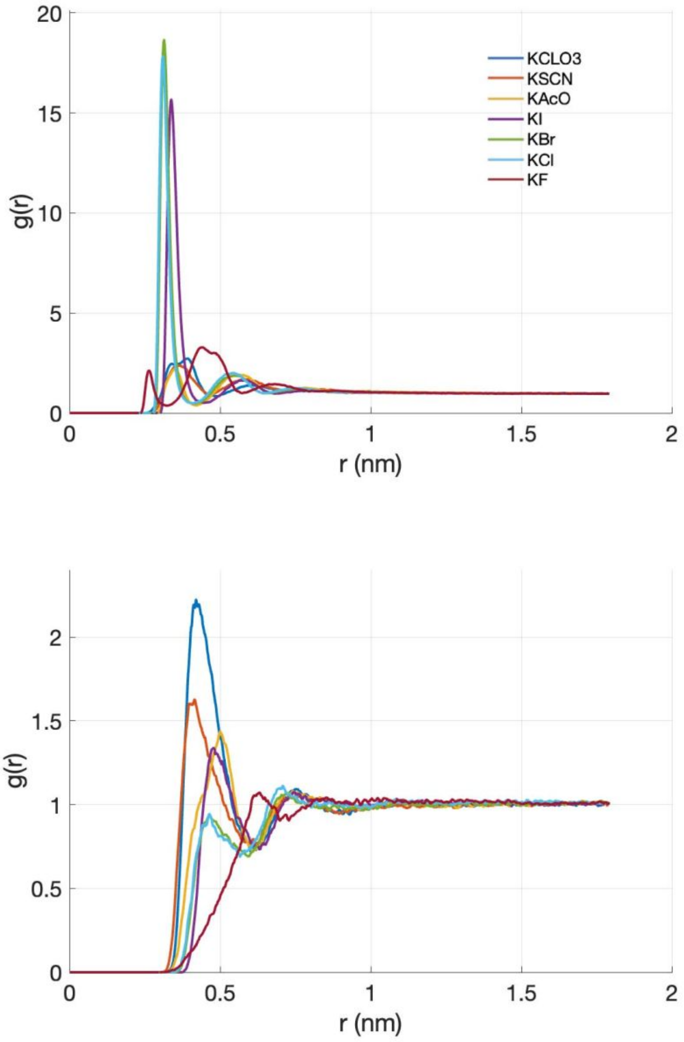

Figure S1: At top RDF cation-anion and at bottom RDF anion-N_2 showing the behaviour at long distances. All RDFs converge to unity at the box limit.

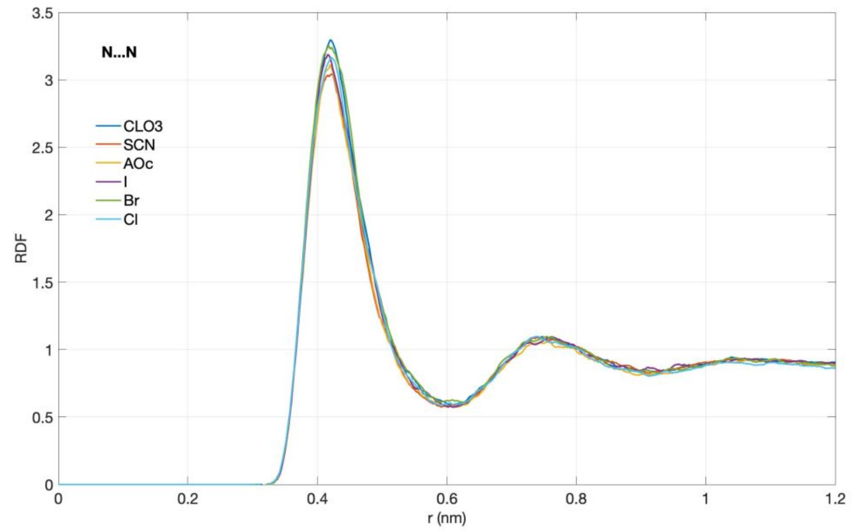

Figure S2: N…N radial distribution functions for all investigated systems. Despite the supersaturated N_2 concentration employed to improve statistical sampling, the RDFs exhibit only a short-range contact peak and rapidly converge to unity at long distances, indicating the absence of N_2 aggregation or clustering. The coordination number (CN) of the N-N pair is ~0.5 for all solutions, confirming that the elevated N_2 content does not lead to significant pair formation. Within statistical uncertainty, the profiles are essentially indistinguishable across systems, demonstrating that the supersaturated conditions do not induce artificial gas structuring in the simulation box.

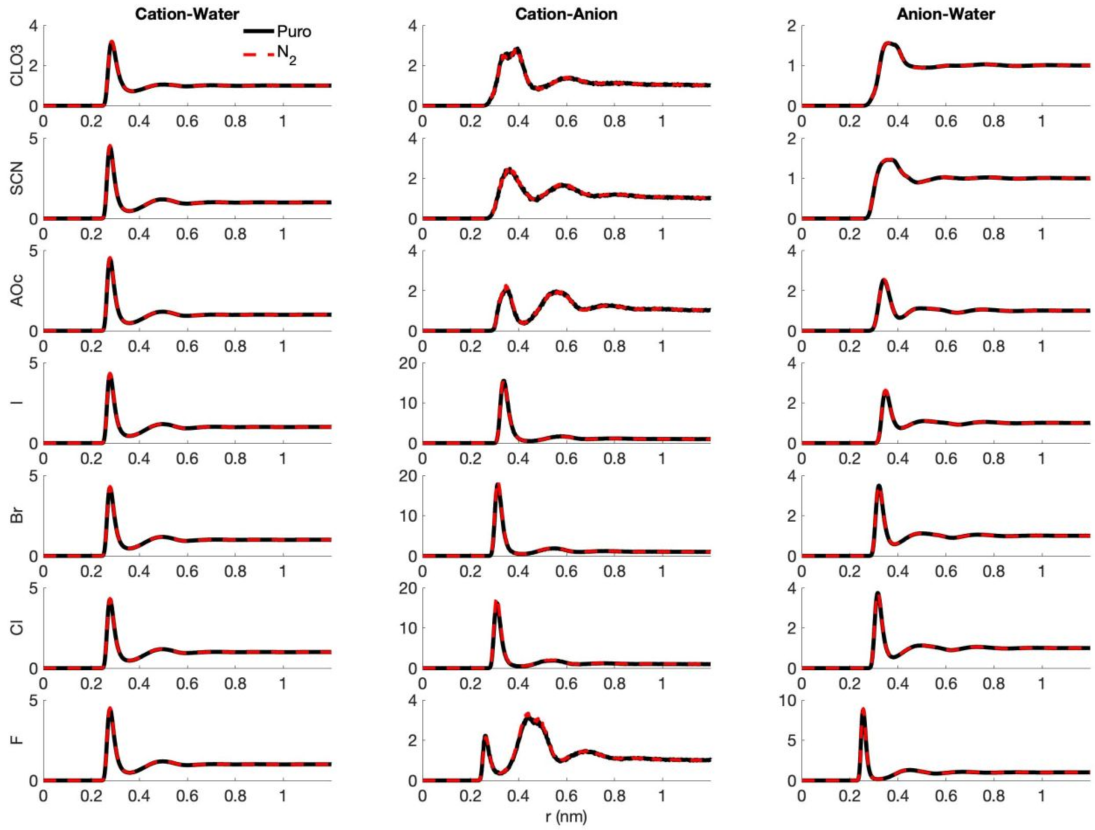

Figure S3: RDF's cation-water, cation-anion and anion-water for all systems investigated comparing the structure with and without N_2 molecules. The comparison make explicit that RDFs are essentially unchanged by the presence of N_2 (within statistical uncertainty).

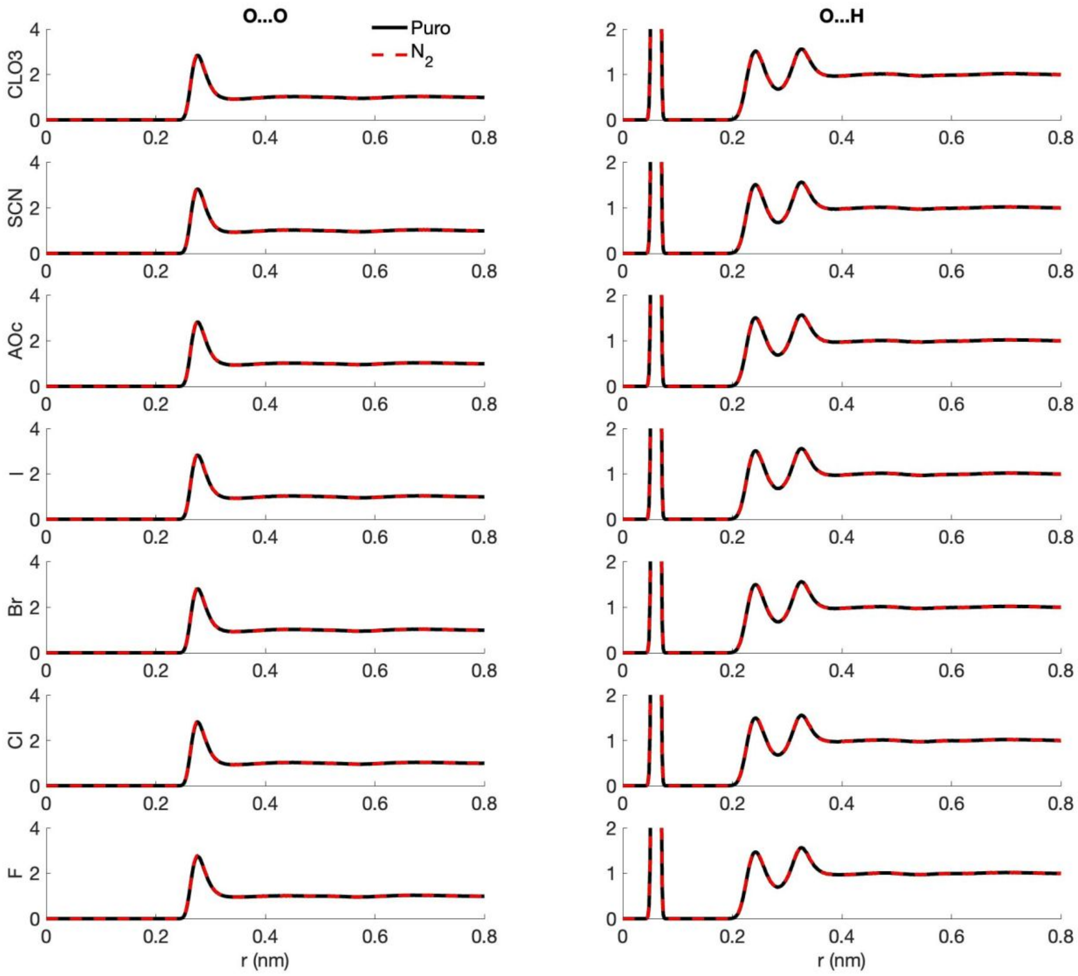

Figure S4: RDF's (O…O and O…H) between water molecules for all systems investigated comparing the HB structure with and without N_2 molecules. The comparison make explicit that RDFs are essentially unchanged by the presence of N_2 (within statistical uncertainty).

Table S2: The table reports diffusion coefficients (D) and ionic conductivity (σ in S m^{-1}). Diffusion coefficients (D em $10^{-5}cm^2/s$) of ions and water in different electrolytes, with and without N_2, were obtained from the slope of the mean square displacement (MSD) via the Einstein relation. Rows labeled $+N_2$ correspond to systems containing dissolved nitrogen. Δ denotes the absolute variation relative to the pure system, with the percentage change in parentheses. Ionic conductivity was estimated using the Nernst-Einstein relation from cation and anion diffusion coefficients. Note that this model assumes independent ionic motion and neglects ionic correlations, ion pairing, and collective effects, potentially overestimating conductivity.

System	D(-) without N_2	D(-) with N_2	D(+) without N_2	D(+) with N_2	σ (sem N_2)	σ (com N_2)
**KAc**	1.5	1.2	2.9	2.6	4.4	3.8
**KF**	1.5	1.6	2.3	2.9	3.8	4.5

The ionic conductivity was estimated using the Nernst–Einstein relation. For 1:1 electrolytes, this expression simplifies to :

$$\sigma = \frac{F^2}{RT}\sum_i z_i^2 \, c_i \, D_i = \frac{F^2}{RT} \, c \, (D_+ + D_-)$$

relating conductivity directly to the sum of cation and anion diffusion coefficients.
